# Comparative Evaluation of Antimicrobial Efficacy and Fluoride Release of Seven Different Glass-Ionomer-Based Restorative Materials

**DOI:** 10.3290/j.ohpd.a44140

**Published:** 2020-07-04

**Authors:** Savas Sagmak, Emrullah Bahsi, Nida Ozcan, Omer Satici

**Affiliations:** a Research Assistant, Dicle University, Faculty of Dentistry, Department of Restorative Dentistry, Diyarbakir, Turkey. Idea, hypothesis, experimental design, performed the experiments in partial fulfilment of requirements for a degree.; b Associate Professor, Dicle University, Faculty of Dentistry, Department of Restorative Dentistry, Diyarbakir, Turkey. Wrote the manuscript, proofread the manuscript.; c Researcher, Dicle University Medical Faculty, Department of Medical Microbiology, Diyarbakır, Turkey. Performed tests.; d Professor, Dicle University Faculıv Medical Faculty, Department of Biostatistics, Diyarbakir, Turkey. Consulted on and performed statistical evaluation.

**Keywords:** fluoride, glass ionomer, antimicrobial, *S. mutans*, *L. acidophylus*

## Abstract

**Purpose:**

The aim of this study was to evaluate one high-viscosity glass-ionomer cement (EQUIA/GC), two resin-modified glass ionomers (Fuji II LC/GC, Photac Fil Quick Aplicap/3M Oral Care), two traditional glass ionomers (Ketac Molar Easymix/3M, Fuji II/GC), and two compomers (Freedom/SDI, Dyract XP/Dentsply Sirona) through a comparison of fluoride release and antimicrobial effects.

**Materials and Methods:**

A total of 210 samples were prepared, as 10 for each of the 7 materials for fluoride release and 20 for each material for the antimicrobial effect tests. To measure fluoride release, 5 ml distilled water and 5 ml TISAB II were added to the samples, which were then incubated at 37˚C. The fluoride levels of the material were measured using the selective ion electrode on days 1, 3, 7, 14 and 28. To compare the antimicrobial effects, 20 samples were divided in two groups and implanted in culture media containing *Streptococcus mutans* and *Lactobacillus acidophylus.* Measurements were taken on days 2, 4 and 6. The diameter of the inhibition zone was recorded in millimetre (mm).

**Results:**

All the materials released fluoride and the difference between them was determined to be statistically significant (p < 0.01). The antimicrobial effect values of the materials against *S. mutans* and *L. acidophylus* were evaluated and statistically significant difference was determined between the materials on all the measurement days.

**Conclusions:**

All the materials were observed to release fluoride. With the exception of the compomers, all the other materials showed an antimicrobial effect against *S. mutans* and* L. acidophylus*.

Dental caries is one of the most widespread chronic diseases that can be seen all over the world in every age group. According to the World Health Organization, three in four of the global population are affected with dental caries rates of 60–90% in school-age children, and almost 100% in adults. Dental caries is therefore an infectious disease, which may results in various levels of pain and discomfort, when various preventive and curative processes are neglected it may lead to serious problems.^23,27^

The restoration of teeth with caries is one of the primary treatment requirements.^5^ Comprehensive research in the field of modern dentistry has led to the development of various restorative materials to meet this requirement.^21^ Over the last hundred years, amalgam was the most commonly used material in dentistry. However, the mercury contained in amalgam is harmful both to human health and the natural environment, so this has increased the research into new restorative materials.^13^

Glass-ionomer cements (GIC) were first produced by Wilson and Kent in 1972. These came into widespread use as they can release fluoride and can chemically bind to hard dental tissues.^5^ A thermal expansion coefficient compatible with hard dental tissues and biocompatibility are advantageous characteristics. The magnitude of the antimicrobial effect of GICs partly depends on the low pH and fluoride release.^37^ The relatively weak mechanical properties are the primary limitations of GICs in clinical applications. With several modifications made to GICs, it became a stronger and more aesthetic material compared to previous conventional GICs.^5,19^

Resin-modified GICs (RMGIC) were produced in the 1980s to replace traditional GICs. The mechanical and physical properties of RMGICs are between those of traditional GIC and resin composites. These new materials are recommended for primary teeth restoration, for permanent teeth in the areas remaining under occlusal function.^20^ Due to the rapid polymerisation property, they can be used in children and patients for whom cooperation is difficult, such as disabled individuals.^26^

Several studies have been conducted to evaluate the success of traditional and RMGIC restorations. However, the results in Class II cavities have generally not been satisfactory.^16,28^ Due to the fragile structure of the material, surrounding support by the hard dental tissues is required. Therefore, more successful results have been obtained in Class I cavities than in Class II.^35^ High-viscosity GICs have been developed to increase the insufficient physical properties of traditional GICs and the resistance to wear against chewing forces.^32^ The ratio of powder to liquid in traditional GICs is 3:1 or 4:1, whereas in high-viscosity GICs this ratio is 6:1 or 7:1.6 The polymerisation mechanisms by way of an acid-base reaction are similar to those of traditional GICs and the bending and compression resistance, surface hardness and resistance to wear of these cements is increased and solubility is decreased.^6,12^

Compomer is a restorative material that is produced by combining the positive characteristics of resin composites and GICs. The main aim in production is to provide a material that can bind well to hard dental tissues without acidification.^4^ Compomers are a widely used material in paediatric dentistry because of their ease of use and physical properties similar to those of resin composites.^2^ Nevertheless, secondary caries of compomers has been reported to be one of the most common causes of loss of restoration. According to literature, 60–70% of repeated restorations originate from secondary caries. Microleakage, which causes secondary caries, leads to the accumulation of bacteria in the restoration borders.^10,21^

Microorganisms play an important role in the onset and progression of dental caries, with *Streptococcus mutans* the primary bacteria species related to the onset. *S.* *mutans* and *Lactobacillus acidophilus* are responsible for the progression of caries and the formation of secondary caries.^10,22^ The ability to prevent secondary caries is an important property of restorative material. Fluoride release from restorative material is extremely important because of the preventive effect against possible caries. There are several dental restorative materials available on the market that contain fluoride such as traditional GIC, RMGIC, compomer and giomer.^25^

Fluoride release is an important property showing the antimicrobial effect of the restorative material. Various factors are associated with the short- and long-term fluoride release from restorative materials, such as the amount and nature of the integrated fluoride, the matrix of the material and the hardening reactions.^10,38^

The aim of this study was to evaluate the fluoride release and antimicrobial efficacy of two traditional GICs (Fuji II and Ketac Molar), two RMGICs (Fuji II LC, Photac Fil), a high-viscosity GIC (Equia) and two compomers (Dyract XP, Freedom).

## Materials and Methods

This study was planned and conducted in Dicle University, Faculty of Dentistry, Department of Restorative Dentistry, and the experiments were completed in a period of 2 months. The study was conducted in two stages; Stage 1: the evaluation of fluoride release from seven different materials at different time intervals, Stage 2: the measurement of antibacterial activity against *S. mutans* and *L. acidophilus* on different days.

The materials tested:

- Group 1: High-viscosity GIC, Equia (GC, Tokyo, Japan)
- Group 2: RMGIC, Fuji II LC (GC, Tokyo, Japan)
- Group 3: RMGIC Photac Fil Quick Aplicap (3M Oral Care, Seefeld, Germany)
- Group 4: Compomer, Dyract (Dentsply Sirona, Bensheim, Germany)
- Group 5: Compomer, Freedom (SDI, Bayswater, Australia)
- Group 6: Traditional GIC, Fuji II (GC, Tokyo, Japan)
- Group 7: Traditional GIC, Ketac Molar (3M Oral Care, Seefeld, Germany)

For each experimental group, 30 sample cylindrical plastic moulds 4 mm deep and 7 mm in diameter were prepared. The samples in Groups 1, 2 and 3 were polymerised with a halogen lamp radiating blue light for periods determined according to the manufacturer’s instructions. After placement of the materials in the moulds, the samples in Groups 4 and 5 were polymerised from above and below for 40 s with a halogen lamp (470 nm wavelength and 75 watt nominal intensity) radiating blue light. The samples for Groups 6 and 7 were prepared by manually mixing with a paper mixing cushion and an agate spatula and the moulds were then filled. Each sample was covered with mylar tape and a glass cover slip and the room temperature was adjusted to 37˚C.

### The Measurement of Fluoride Release

**Fig 1:**
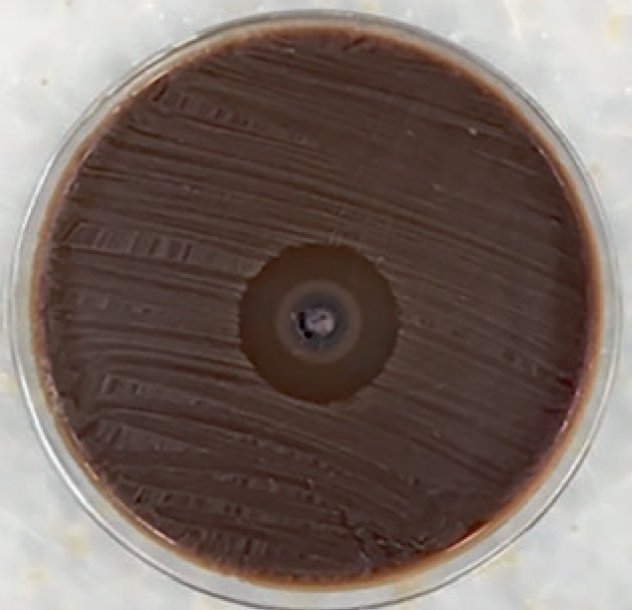
Antimicrobial activity of chlorhexidine against to *S. mutans* (measurement of second day).

The analyses were made using an Orion Fluoride electrode (9609 BNWP) in a Thermo Orion 720 A+ ionometer (Fig 1), and 0.1 MF standard (Orion Ionplus application solution 940906, ThermoFisher Scientific, Waltham, MA, USA) special filling fluid (Orion Ionplus filling solution 900061) placed within the fluoride electrode (standard methods 2005 21st ed. 4550 F-C). In the fluoride ion analysis with the electrode, TISAB II solution (Orion Ionplus application solution 940909) was used as a buffer preventing entry. For the calibration of the electrode, four calibration standards (1–10–50–100 ppm) were used according to the concentration range to be examined. The electrode was immersed into the cup with the sample, distilled water and TISAB II at the end of days 1, 3, 7, 14 and 30, the reading was taken, and the value seen on the screen was recorded. Before and after each measurement, the tip of the electrode was washed in distilled water and lightly dried to remove any remnants of fluoride ion.

### Evaluation of Antibacterial Activity

For each experimental group, 20 samples were tested with *S. mutans* ATCC 25175 and *Lactobacillus acidophilus* ATCC 11975 standard bacteria isolates. The agar diffusion test was used to evaluate the antibacterial effect. Sheep blood agar (ThermoScientific Oxoid, Basingstoke, UK) and Man, Ragosa and Sharpe (MRS) media were used for the agar diffusion test. Immediately following dilution with 0.5 ml tripticase soy broth and 4 h of incubation, the lyophilised *S. mutans* isolate was densely seeded in the 5% sheep blood agar medium. Immediately following dilution with 0.5 ml MRS broth and 4 h of incubation, the lyophilised *L. acidophilus* isolate was densely seeded in the MRS agar solid medium. Using the blind end of a micropipette, wells 7 mm in diameter were made in the agar plate. The samples of the experimental groups were prepared according to the manufacturer’s instructions and placed in the wells. The sets of wells were completely filled with the samples. There was a control group (chlorhexidine 2%). The culture plates were incubated at 37˚C. On days 2, 4 and 6, the diameter of the inhibition zone was recorded in mm. The greatest distance between two points was measured from the outer border of the inhibition zone formed around the wells.

### Statistical Analysis

Statistical analyses of the study data were made using SPSS vn 24 software. Descriptive statistical methods were used (mean, standard deviation, median, interquartile range). In the five repeated measurements of the seven different groups, the Friedman test was used, and for the separate comparisons of the groups, the Kruskal–Wallis test. The Mann-Whitney U test was applied in the paired comparisons when a difference was observed between the groups. The Pearson r correlation coefficient was used in the calculation of the correlations between the continuous measurement variables. A value of p < 0.05 was accepted as statistically significant.

## Results

In the comparison of the mean fluoride release amounts, the highest value of fluoride release was determined in the Fuji II LC compared to the other materials. The amounts of fluoride release at the different time points and the mean values are shown in Table 1. Freedom was determined to be the material with the lowest fluoride release. The differences between the materials in respect of fluoride release on all the measured days were found to be statistically significant (p < 0.05).

**Table 1:** Fluoride release at different days

Groups	Intergroup comparisons for fluoride release (mean ± SD, mg/lt)					
	Day 1	Day 3	Day 7	Day 14	Day 28	Mean
Equia	12.66 ± 0.98	83.53 ± 2.52	40.27 ± 6.17	35.91 ± 1.06	35.59 ± 0.85	41.59 ± 0.57
Photac fill	39.27 ± 5.91	63.13 ± 2.63	52.83 ± 7.11	42.27 ± 6.21	35.67 ± 4.68	46.63 ± 2.78*
Fuji II LC	19.08 ± 4.62	48.27 ± 7.70	67.98 ± 2.50	61.86 ± 2.10	47.50 ± 1.89	48.93 ± 2.26*
Freedom	1.40 ± 0.12	2.52 ± 0.19	3.17 ± 0.22	3.85 ± 0.25	5.15 ± 0.33	3.22 ± 0.19
Dyract XP	7.42 ± 2.78	17.56 ± 3.49	23.53 ± 3.75	31.62 ± 3.89	43.92 ± 2.99	24.81 ± 2.97
Ketac Molar	32.57 ± 1.33	26.53 ± 0.90	24.36 ± 0.45	22.41 ± 0.41	22.17 ± 0.48	25.60 ± 0.33
Fuji II	48.14 ± 5.08	40.06 ± 6.17	34.53 ± 3.56	31.46 ± 3.29	30.46 ± 2.53	36.93 ± 2.13
Kruskal– Wallis	Ki kare = 65.95 p = 0.000	Ki kare = 66.73 p = 0.000	Ki kare = 66.47 p = 0.000	Ki kare = 63.33 p = 0.000	Ki kare = 64.10 p = 0.000	Ki kare = 65. 67 p = 0.000

* Paired comparisons between all groups were statistically significant (p < 0.05) except Photac fill–Fuji II LC groups (p > 0.05).

When the antimicrobial effect against *S. mutans* was examined, the highest effect of the general mean value of all the measurements was seen in the control group (chlorhexidine), followed by Fuji II LC, Equia, Photacfil, Fuji II, and Ketac Molar Easymix, respectively. The antimicrobial effect values against *S. mutans* are shown in Table 2. The antimicrobial effect of chlorhexidine against *S. mutans* on measurement day 2 is shown in Figure 1. Dyract XP and Freedom showed no antimicrobial effect. The differences between the groups on all the measurement days were determined to be statistically significant (p < 0.05).

**Table 2:** Antimicrobial activity values against *S. mutans* according to measurement days

Groups	Antimicrobial activity against *S. mutans* (mm)			
	Day 2	Day 4	Day 6	Mean
Equıa	15.85 ± 1.21	15.16 ± 1.36	14.83 ± 1.52	15.28 ± 1.33
Photac fill	13.04 ± 0.54	11.73 ± 0.86	10.97 ± 1.18	11.91 ± 0.74*
Fuji II LC	17.25 ± 0.67	16.12 ± 0.60	15.58 ± 0.77	16.31 ± 0.74
Freedom	7.00 ± 0.00	7.00 ± 0.00	7.00 ± 0.00	7.00 ± 0.0**
Dyract XP	7.00 ± 0.00	7.00 ± 0.00	7.00 ± 0.00	7.00 ± 0.0**
Ketac Molar	13.16 ± 0.66	11.50 ± 0.85	10.30 ± 0.95	11.65 ± 0.64*
Fuji II	12.84 ± 0.40	12.19 ± 0.17	10.48 ± 0.78	11.83 ± 0.28*
Chlorhexidene	24.19 ± 0.39	23.70 ± 0.44	22.72 ± 0.65	23.53 ± 0.42
Kruskal–Wallis	Chi-square = 62.28 p = 0.000	Chi-square = 62.62 p = 0.000	Chi-square = 62.52 p = 0.000	Chi-square = 62.37 p = 0.000

* Paired comparisons between all groups were statistically significant (p < 0.05) except Photac fill–Ketac Molar. Photac fill–Fuji II and Ketac Molar–Fuji II groups (p > 0.05). ** Paired comparisons between all groups were statistically significant (p < 0.05) except Freedom–Dyract XP groups (p > 0.05).

**Fig 2:**
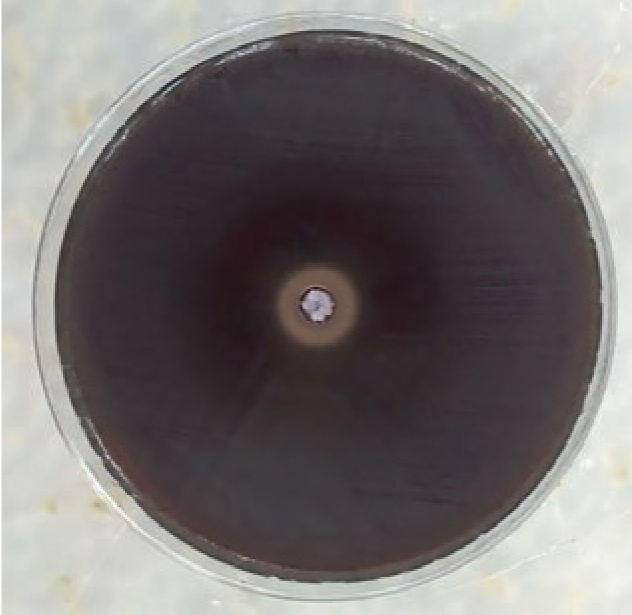
Antimicrobial activity of chlorhexidine against to *L. acidophylus* (measurement of second day).

In the examination of antimicrobial activity against *L. acidophilus*, the greatest effect was seen in the control group (chlorhexidine) in the general mean values of all the measurements. This was followed by Fuji II LC, Fuji II, Equia, Photac Fil, and Ketac Molar Easymix, respectively. The antimicrobial effect values against* L. acidophilus* are shown in Table 3. The antimicrobial effect of chlorhexidine against *L. acidophilus* on measurement day 2 is shown in Figure 2. Dyract XP and Freedom showed no antimicrobial effect. The differences between the groups on all the measurement days were determined to be statistically significant (p <0.05).

**Table 3:** Antimicrobial activity values against *L. acidophylus* according to measurement days

Groups	Antimicrobial activity against *L. acidophylus* (mm)			
	Day 2	Day 4	Day 6	Mean
Equıa	13.36 ± 1.17	11.52 ± 0.50	10.69 ± 0.51	11.85 ± 0.49*
Photac fill	10.50 ± 0.52	8.95 ± 0.31	8.58 ± 0.41	9.34 ± 0.27
Fuji II LC	16.37 ± 0.97	10.28 ± 1.35	9.51 ± 0.71	12.05 ± 0.90*
Freedom sdı	7.00 ± 0.00	7.00 ± 0.00	7.00 ± 0.00	7.00 ± 0.00**
Dyract XP	7.00 ± 0.00	7.00 ± 0.00	7.00 ± 0.00	7.00 ± 0.00**
Ketac Molar	9.07 ± 0.67	7.34 ± 0.22	7.02 ± 0.04	7.81 ± 0.21
Fuji II	12.23 ± 0.39	11.86 ± 0.40	11.71 ± 0.41	11.93 ± 0.37*
Chlorhexidene	19.85 ± 0.84	19.08 ± 0.55	17.66 ± 1.39	18.86 ± 0.78
Kruskal–Wallis	Chi-square = 67.321 p = 0.000	Chi-square = 65.42 p = 0.000	Chi-square = 65.69 p = 0.000	Chi-square = 63.11 p = 0.000

* Paired comparisons between all groups were statistically significant (p < 0.05) except Equia–Fuji II LC, Equia–Fuji II and Fuji II LC–Fuji II groups (p > 0.05). ** Paired comparisons between all groups were statistically significant (p < 0.05) except Freedom–Dyract XP groups (p > 0.05).

## Discussion

In this study, the values were determined of antimicrobial activity and the fluoride release of current restorative materials containing glass ionomer, which are used in dentistry. Equia, Fuji II LC, Photacfil, Fuji II, Ketac Molar Easymix, Dyract XP and Freedom materials were evaluated in the study. There are very few previous studies in literature related to the fluoride release of Equia material.

In the determination of fluoride release values, an ion analyser and specific fluoride electrode are used.^9^ In this study, the Orion 720A+ device and the Orion Fluoride electrode were used. To correct the pH of the solution and to ionise the fluoride in the fluid, it has been reported that it is necessary to add certain concentrations of TISAB solution to the solution.^11,34^ In the current study, a 5% concentration of TISAB II solution was added to the deionised water immediately before the measurements. It has also been reported that the electrode must be calibrated immediately before the measurement of fluoride release values by preparing solutions at concentrations different from those of standard fluoride solutions.^11,31^ In the current study, the standard fluoride solutions for calibration were freshly prepared on each measurement day. Calibration of the device was made by repeating with specific ranges on each measurement day.

The amount of fluoride released from GICs depends on several factors. Intrinsic factors such as formulation, solubility and porosity affect the amount of fluoride release from the material.^38^ The fluoride content and expression should be at the maximum level without creating a negative effect on the physical and mechanical properties of the material.^14^ In the current study, the maximum fluoride release was observed from Fuji II LC and the minimum from Freedom.

Rao et al measured fluoride release in two traditional GICs (Fuji VII, Fuji II), an RMGIC (Fuji II LC), a composite (Tetric Ceram) and a compomer (F2000) on the 1st, 7th and 28th days. The highest amount of fluoride release was reported to be from the traditional GICs, Fuji II and Fuji VII, followed by Fuji II LC, F2000 and Tetric Ceram, respectively, and the samples prepared with compomer and composite could not be reloaded with fluoride.^30^ In our study, Fuji II LC produced a greater amount of fluoride release than Fuji II. That a high amount of fluoride release was observed from Fuji II LC could be related to the chemical content and form of mixing of the material.

The compomers had a low amount of fluoride release, in relation to the GIC and RMGIC groups. This finding was similar previous studies.^8^ The difference between GICs and compomers can be attributed to the contact with water before and after polymerisation. The porosity of the GIC and denseness of the polymerised compomer play the largest role. Therefore, the infiltration/absorbance capacity of the GIC versus the compomer are different. The GIC also has free fluoride ions in the matrix that allow much faster diffusion from the GIC vs the compomer. In a study conducted over 36 months, it was reported that the highest fluoride release was from GICs in the first year, but in the second year the amounts were equal.^1^

Garoushi et al compared fluoride release values throughout 10 days using Dyract XP (compomer), Compglass F (compomer), ACTIVA-Restorative (Bioactive composite), BEAUTIFIL-II (Giomer) and GC Fuji II LC (RMGIC). On all the measurement days, GC Fuji II LC showed the highest fluoride release and BEAUTIFUL-II, the lowest. The difference between the materials was determined to be statistically significant.^15^ Also in our study, while Fuji II LC showed the highest amount of fluoride release, Dyract XP released a very low amount of fluoride.

Dasgupta et al compared fluoride release values on days 1, 3, 7, 14, 21 and 28 using GP IX Extra (RMGIC), EQUIA Forte Fil (high-viscosity GIC), Beautifil Bulk (Bulk fil), Dyract XP (Compomer), Tetric N-Ceram (Composite). On all the measurement days, EQUIA Forte Fil showed the highest fluoride release and Tetric N-Ceram, the lowest. The difference between the materials was determined to be statistically significant.^8^ Also in the present study, Dyract XP released a lower amount of fluoride than high-viscosity glass-ionomer cement and resin-modified GIC.

The results of the current study showed that the traditional GICs released the most fluoride on the 1st day. This result was similar to the findings of previous studies.^25,29^ In a study by Mousavinasab et al, they observed the highest fluoride release from Fuji VII and Fuji IX on the first day. This initial ‘burst effect’ of fluoride removes bacteria which may be found in the cavity and has an effect on the remineralisation of enamel and dentine.^38^

The antimicrobial effect of cements is related to the low pH at the start of the hardening reaction, fluoride release and the antimicrobial components. Various ions such as fluoride that are released by GICs provide the antimicrobial and protective properties against potential caries. Fluoride is accepted as the basic factor in the antimicrobial activity of these cements.^7^

Fluoride inhibits the production of organic acid and glucan of *S. mutans*, which is the primary aetiological factor of caries lesions. Therefore, *S. mutans* is routinely used in antimicrobial activity tests.^17^ In the current study, the agar diffusion method was used to evaluate antimicrobial activity. This method is a simple and inexpensive method in the routine examination of bacteria resistance.

The use of appropriate agar in the agar diffusion test is extremely important for both the development level of the test microorganisms and in respect of the effect on the size of the microorganisms in the medium. In previous studies, bacteria strains have been prepared in Tryptic Soy Blood Agar, Mitis Salivaris Agar, Müller-Hinton Agar, Brain Heart Infusion Agar and incubated at 37˚C for different periods.^3,24^ In the current study, *S. mutans* isolate was seeded in sheep blood agar (ThermoScientific Oxoid, Basingstoke, UK) medium and lyophilised *L. acidophilus* isolate was seeded in MRS medium.

In a study by Tiwari et al the antimicrobial effects were compared of Fuji II (traditional GIC), Fuji IX (GIC), Compoglass F (compomer) and Zirconomer (GIC strengthened with zirconium) and the greatest effect was seen in Zirconomer followed by Fuji IX, whereas Compoglass F showed no antimicrobial effect.^36^ In the current study, the Fuji II LC showed a greater effect than the traditional GICs and no antimicrobial effect was observed in the compomers for both bacterial strains.

In a study by Kramer et al the effect of fluoride on demineralisation was examined on 124 extracted 3rd molar teeth. Grade V cavities were opened and filled with a traditional GIC (Ketac Molar), two RMGICs (Photac Fil, Ketac N100 3M Oral Care) and a resin composite as a control group (Filtek Supreme XTE), then left exposed to *S. mutans* for 10 days. When sections taken from the teeth were examined, the GICs and RMGICs were seen to have provided greater protection against demineralisation and secondary caries than resin composite.^18^ In our study, Photac Fil and Ketac Molar showed similar effects against *S. mutans*.

Shashibhushan et al compared antimicrobial activity against *S. mutans* using Fuji II (traditional GIC), Fuji IX (traditional GIC) and Fuji II LC (RMGIC) materials. The highest effect was shown by Fuji II LC, followed by Fuji II and Fuji IX, respectively.^33^ The results of the current study showed that Fuji II LC had a greater antimicrobial effect than Fuji II.

The largest inhibition zone diameter was shown by Fuji II LC and the smallest by Dyract XP and Freedom. This difference between the materials can be attributed to the difference in fluoride release.^36^

## Conclusions

In the light of the data obtained in this study, it can be said that fluoride release is effective in the prevention of caries formation by reducing primary caries bacteria. High-viscosity GICs and RMGICs can be used in deep dentine cavities that are exposed to the interproximal areas where bacterial accumulation and microleakage occur more readily. The use of compomer is not appropriate in patients with widespread caries, where the caries risk was not appropriately reduced by dental health education and instructions. However, there is a need for long-term clinical studies and new findings to support the results of this in vitro study.
